# Clinic-based ultra-wide field retinal imaging in a pediatric population

**DOI:** 10.1186/s40942-019-0171-1

**Published:** 2019-12-12

**Authors:** Nikisha Kothari, Stacy Pineles, David Sarraf, Federico Velez, Gad Heilweil, Gary Holland, Colin A. McCannel, Tania Onclinx, Tara A. McCannel, SriniVas R. Sadda, Steven D. Schwartz, Irena Tsui

**Affiliations:** Department of Ophthalmology, Stein Eye Institute, Doheny Eye Institute, University of California, Los Angeles, 100 Stein Plaza, Los Angeles, CA 90095 USA

**Keywords:** Wide-field imaging, Pediatric imaging, Fluorescein angiography

## Abstract

**Background:**

Pediatric retinal disorders, although uncommon, can be challenging to assess in the clinic setting and often requires an exam under anesthesia. The purpose of our study was to evaluate the use of ultra-wide field retinal imaging in children without sedation in an outpatient clinic.

**Methods:**

We performed a retrospective case series of patients 18 years or younger who received ultra-wide field imaging over a one year period. The age, gender, and clinical course were documented. Color fundus and red-free images were reviewed to assess field of view. Ultra-wide field autofluorescence (UWF-FAF) was evaluated for abnormal autofluorescence patterns and ultra-wide field fluorescein angiography (UWF-FA) was assessed for angiographic phase and field of view.

**Results:**

A total of 107 eyes of 55 patients with a mean age of 11.1 years (SD 3.7 years, range 3–18 years) were evaluated. Twenty-seven (49%) patients were male. The most common diagnosis was retinopathy of prematurity (7 of 55 patients, 12.7%) followed by trauma (7.4%), Coats disease (7.4%), and rhegmatogenous retinal detachment (7.4%). The number of quadrants visualized anterior to the equator correlated with patient age (r = 0.4, *p* < 0.01). On UWF-FA, 6 of 14 patients (43%) had images of the arterial phase captured and 14 of 14 patients (100%) had images of the venous phase or later captured.

**Conclusions:**

We demonstrated that UWF imaging is obtainable in children as young as 3 years old without sedation. UWF fundus photography, UWF-FAF and UWF-FA were useful clinical adjuvants to examination and provide additional information for documenting and monitoring pediatric retinal diseases.

## Background

Pediatric retinal disorders evaluated in the clinic setting include retinopathy of prematurity (ROP), Coats disease, vitreoretinopathies, hamartomas, congenital dystrophies and trauma [1–3]. Obtaining fundus imaging in pediatric patients in the clinic is challenging requiring patient cooperation and a skilled photographer. The RetCam (Clarity Medical System, Pleasanton, CA) is commonly used to manage ROP in the neonatal intensive care unit, and in this setting usually does not require anesthesia because infants are small enough to be swaddled and held still [4, 5]. However, it is a contact camera with a bright light and therefore, in non-infants requires an examination under anesthesia (EUA). Although relatively safe, there are potential risks associated with general anesthesia in children, such as cardiac arrest and long-term neurologic affects [6, 7].

Ultra-wide field (UWF) imaging is a non-contact, non-invasive imaging modality capturing up to 200 degrees of visible fundus that has become essential for understanding and managing peripheral retinal pathology of disease such as diabetes and retinal vein occlusions in adults [3, 8]. The ease and speed of use of non-contact UWF imaging are particularly useful in the evaluation of pediatric retinal disorders [2, 3]. UWF fundus photography can complement physical examination and provide an objective measure to monitor progression. The image field, quality, and resolution obtained during a single exposure is another advantage.

In the setting of pediatric retinal disease, UWF fluorescein angiography (UWF-FA) has broadened our understanding of retinal vascular disorders by delineating the extent of ischemia or peripheral leakage. In several case reports, UWF-FA assisted in the management of retinal vascular diseases such as Coats disease, familial exudative vitreoretinopathy (FEVR), and incontinentia pigmenti syndrome [2, 9–12]. Use of UWF imaging while placing the newborn or infant in an upright position using the “flying baby technique” for evaluating ROP has been described but is not widely used [13–15].

Our group has previously reported on the use UWF-FA in the pediatric population without sedation [2]. The purpose of this study was to evaluate the utilization of UWF imaging including fundus photography, autofluorescence, and fluorescein angiography a variety of disease conditions impacting the pediatric population in a real-world setting. To our knowledge, this is the largest study of evaluating UWF fundus photography in pediatric patients. We postulate that the use of UWF imaging may be increasing as a meaningful clinical adjuvant in the management of pediatric retinal disorders in the clinic setting.

## Methods

With Institutional Review Board approval, a retrospective chart review was done at the Stein Eye Institute, UCLA. An UWF imaging database was searched for patients who had undergone UWF-FA (Optos PLC) at age 18 years or younger from July 1, 2017 to June 30, 2018. All patients that had not previously been imaged were included. Eyes were excluded if poor quality images were obtained due to significant media opacity such as cataract, vitreous hemorrhage, band keratopathy.

Charts were reviewed and data were collected, including age, gender, dilation, diagnosis, and patient course. Patients were imaged based on the physician’s discretion.

### Retinal image acquisition

All patients were dilated with tropicamide 1% and phenylephrine 2.5%. Room lights were dimmed at photographers’ discretion. Patients were imaged sitting on an adjustable height rolling chair with both feet flat on the floor, if possible. A footstool was used when necessary to allow maximum stability. Small children were allowed to sit on a parent’s lap if preferred. If so, then the parent was similarly seated on an adjustable rolling chair with both feet flat on the floor and assisted with positioning the child’s head on the chin rest with the forehead against the upper bar. The photographers instructed the child to look at the fixation target. Eyelid speculums were not used; however manual retraction of the eyelids was performed if needed. Multiple images were acquired, if needed, to capture as many quadrants as possible. Images that were clearly and sharply captured were transferred to electronic medical record at the photographers’ discretion.

Fluorescein angiography was performed with 5% fluorescein dye dosed by weight (7.7 mg/kg or the 35 mg/10 lbs) injected into the patient’s arm. A standardized protocol originally designed for adult patients was used and included a 25-gauge butterfly needle [16].

### Image grading

UWF fundus photos, autofluorescence (UWF-FAF), and UWF-FA were reviewed with Vantage V2 Software (Optos PLC, Dunfermline, Scotland, United Kingdom) and manipulated with standard digital enhancement techniques such as zoom and contrast.

**Fig. 1:**
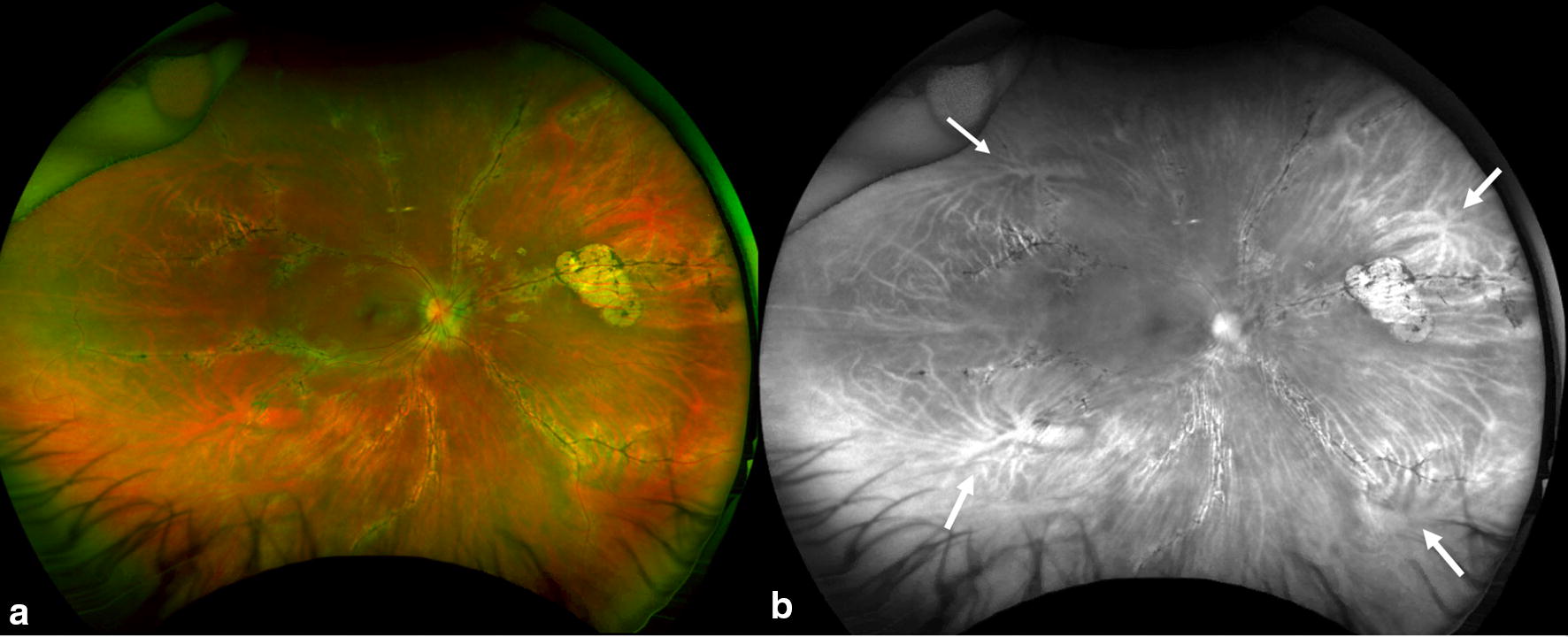
Color and red free fundus photography in pigmented paravenous chorioretinal atrophy. **a** The color photograph demonstrates visualization of most quadrants with lash artifacts inferiorly and nose artifacts temporally in a patient. **b** The red free photographs help illustrate the vortex veins (white arrows) to define the equator. Quadrants visualized were defined based on visualization posterior to the equator

Fundus photographs were evaluated for field of view. Quadrants (superior, nasal, inferior, temporal) were considered visible if fundus until the equator could be evaluated in the images. The equator was defined by the region posterior to the vortex veins, evaluated on composite red-free photographs (Fig. 1). If all quadrants were not captured in one image, the total number of quadrants visualized over serial photographs were tallied and recorded. Statistical analysis correlating patient age with field of view was performed using Pearson bivariate correlation analysis and a student *t* test.

**Fig. 2:**
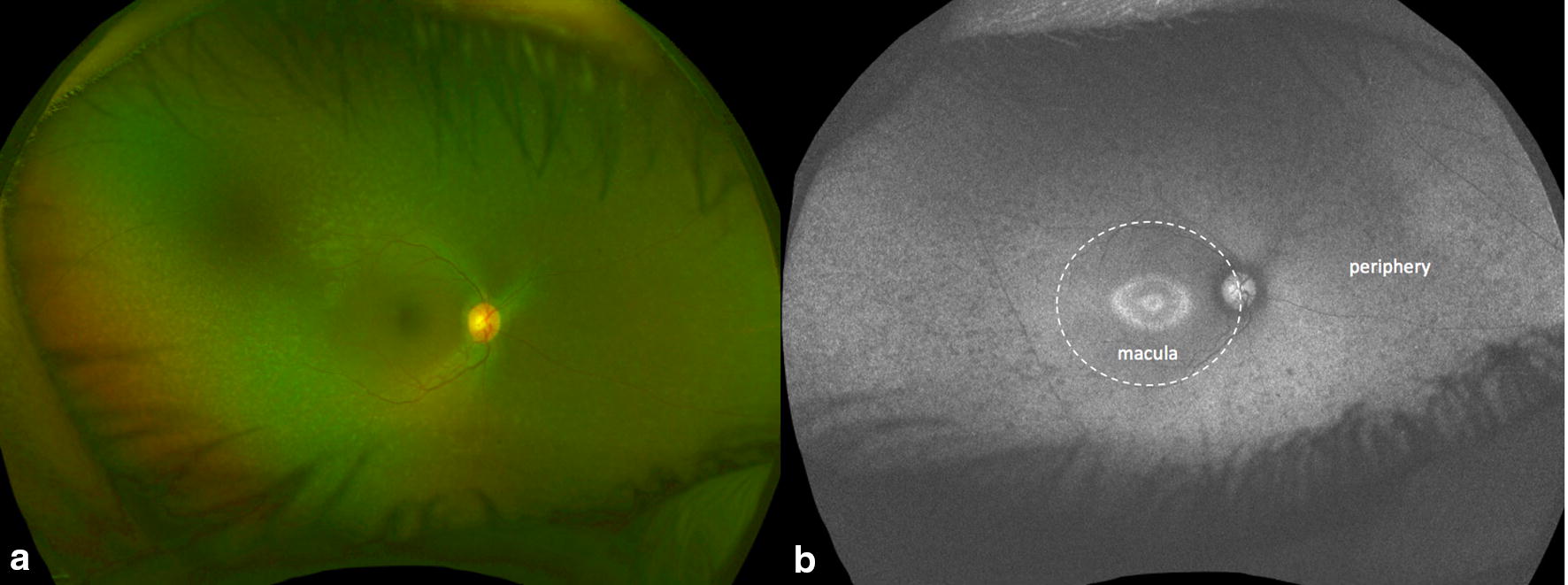
Color and autofluorescence fundus photography in a cone-rod dystrophy. **a** The color photograph demonstrates hypopigmentary specks in the mid-peripheral retina. **b** The fundus autofluorescence demonstrates a ring of hypo and hyperautofluorescence (Robson–Holder ring) and hypoautofluoresent flecks in the mid-periphery

Autofluorescence images were evaluated for presence of any areas of hypo-autofluorescence or hyper-autofluorescence in the macula defined as the area within the arcade vessels and in the periphery defined as outside the arcade vessels or both (Fig. 2).

Fluorescein angiography images were evaluated for the capture of angiographic phases (choroidal, arterial, venous, recirculation). The images were reviewed to evaluate for artifacts during each phase of the angiography. In addition, the number of quadrants visualized from all images of a particular angiographic phase were documented.

## Results

During this one year time span, 107 eyes of 55 patients ages 18 years or younger were imaged using UWF imaging. The mean age was 11.1 years (SD 3.7 years, range 3–18 years). Twenty-seven patients were male (49%). All patients except 3 (94%) had both eyes imaged with dilation. Only the contralateral eye of a patient with Coats disease was imaged; the affected eye had band keratopathy following retinal detachment repair with silicone oil. The other 2 patients did not have dilation or examination of the unaffected eye on the day of imaging, however previous examination revealed a normal fundoscopic exam.

The most common quadrant to be obscured by artifact was the inferior quadrant (19% of eyes) due to superior lashes. The number of quadrants visualized positively correlated with age (r = 0.4, *p* < 0.01). The average age of patients with all quadrants imaged in both eyes (12 years, SD 3.15 years) was significantly older than patients with less than three quadrants imaged in either eye (6.9 years, SD 3.60 years, *p* < 0.01) The youngest patients in the study, one patient aged 3 years and 3 patients aged 4 years, were in this group. In the 8 eyes of these 4 patients, an average of 2.4 quadrants were visualized with the inferior quadrant only captured in one eye. Notably 2 patients with nystagmus were successfully imaged; one patient with nystagmus had all 4 quadrants visualized in both eyes.

**Fig. 3:**
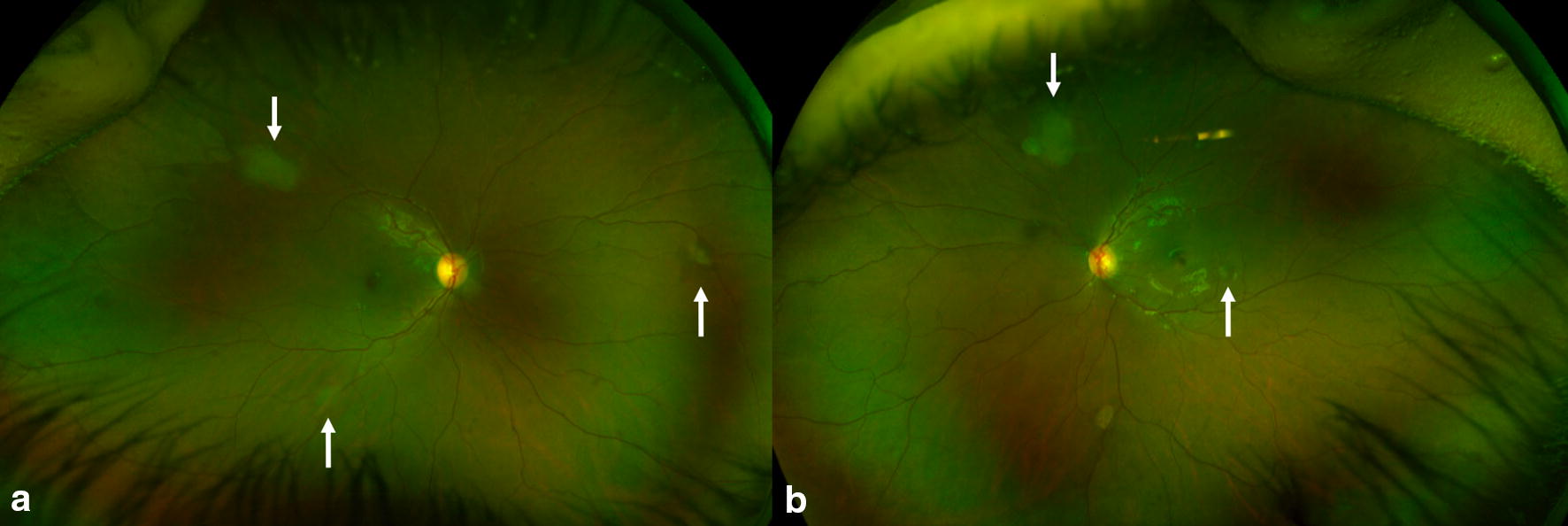
Color fundus photography demonstrating multiple retinal astrocytomas (white arrows) in both eyes of a patient with tuberous sclerosis. **a** Right eye, **b** left eye

The range of indications for obtaining imaging is outlined in Table 1. The most common indication for obtaining imaging was ROP (12.7%). Several rare entities such as pigmented paravenous chorioretinal atrophy and retinal astrocytoma were captured using UWF fundus photography (Figs. 1, 3).

**Table 1:** Indications for ultra-wide field imaging in our pediatric patients

Indication	
Retinal vascular diseases	15
Retinopathy of prematurity	7
Coats disease	4
Incontinenti pigmenti syndrome	2
Familial exudative vitreoretinopathy	1
Branched retinal artery occlusion	1
Peripheral retinal diseases	7
Rhegmatogenous retinal detachment	4
Stickler’s syndrome	1
Myopia/lattice degeneration	2
Uveitis	5
Juvenile idiopathic arthritis	2
Long term use of plaquenil	1
Multifocal choroiditis	1
Blau’s syndrome	1
Pigmentary lesions/tumors	7
Congenital hypertrophy of the retinal pigment epithelium	3
Choroidal nevus	1
Choroidal melanoma	1
Radiation retinopathy (s/p plaque for iris melanoma)	1
Tuberous sclerosis	1
Hereditary diseases	5
Achromatopsia	1
Best vitelliform dystrophy	2
Long-chain 3-hydroxyacyl-CoA dehydrogenase retinal dystrophy	1
Stargardt disease	1
Trauma	4
Other	12
Type 1 diabetes mellitus	2
Visual disturbance/floaters	3
Aphakia	1
Nevus of ota w/o uveal melanoma	1
Bell’s palsy	1
Idiopathic intracranial hypertension	1
Pigmented paravenous chorioretinal atrophy	1
Optic disc hemorrhage	1
Central serous chorioretinopathy	1

UWF-FAF was obtained in forty-eight (87%) patients and abnormal FAF findings were detected in 24 patients (50%). The location of abnormal autofluorescence was as follows: macular only in 1 patient (4%), peripheral only in 7 patients (29%), and both macular and peripheral in 16 patients (67%).

**Fig. 4:**
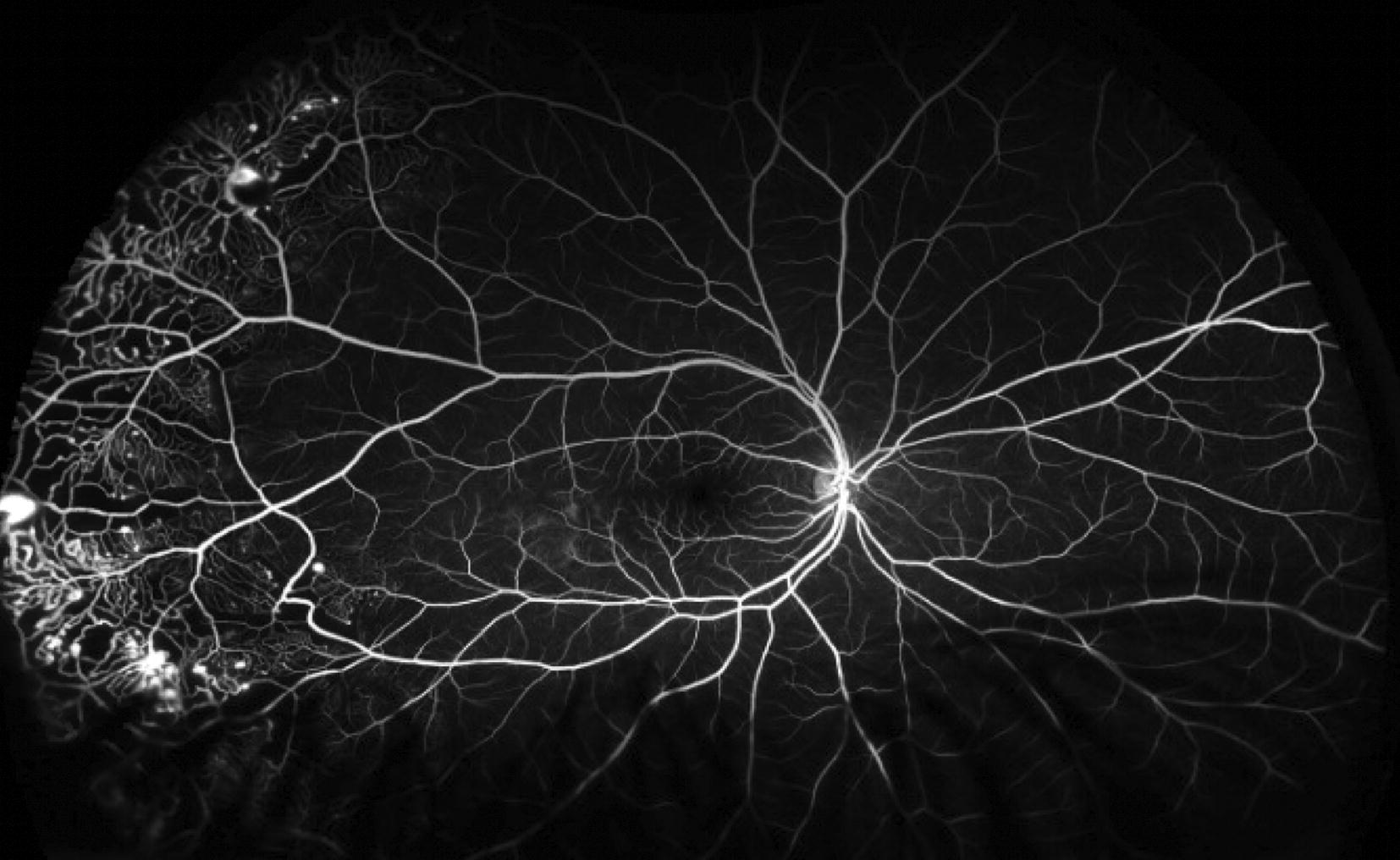
Ultra-wide field fluorescein angiography demonstrates peripheral telengectatic vessels, nonperfusion, and leakage in Coats disease. The patient subsequently underwent an EUA with targeted panretinal photocoagulation

UWF-FA was obtained in 14 (25%) patients and abnormal FA findings were detected in 12 patients (86%). In the patients who received fluorescein angiography, the angiographic phases were evaluated (Table 2). None of the patients had capture of early choroidal filling (0%). Six of the 14 patients (43%) had capture of the arterial phase. All patients (100%) had capture of the venous and recirculation phases. The average visible quadrants ranged from 3.7 to 4 quadrants. An example of a patient who received an UWF-FA with Coats disease in clinic is shown in Fig. 4.

**Table 2:** Angiographic Phase, Number (%) of Patients and Mean Number of Visible Quadrants in That Phase

Angiographic phase	Number of patients	Quadrants visible
Choroidal	0	n/a
Arterial	6 (42.9%)	4
Venous	14 (100%)	3.7
Recirculation	14 (100%)	3.7

**Fig. 5:**
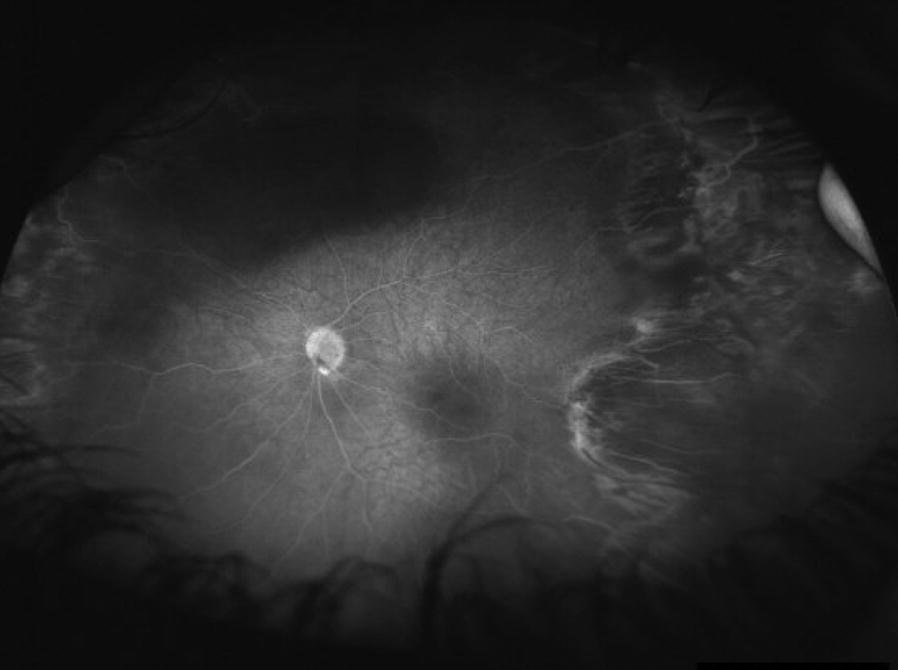
In a patient with retinopathy of prematurity previously treated with laser ablation, ultra-wide field fluorescein angiography demonstrates no leakage. Notably there is temporal dragging

**Fig. 6:**
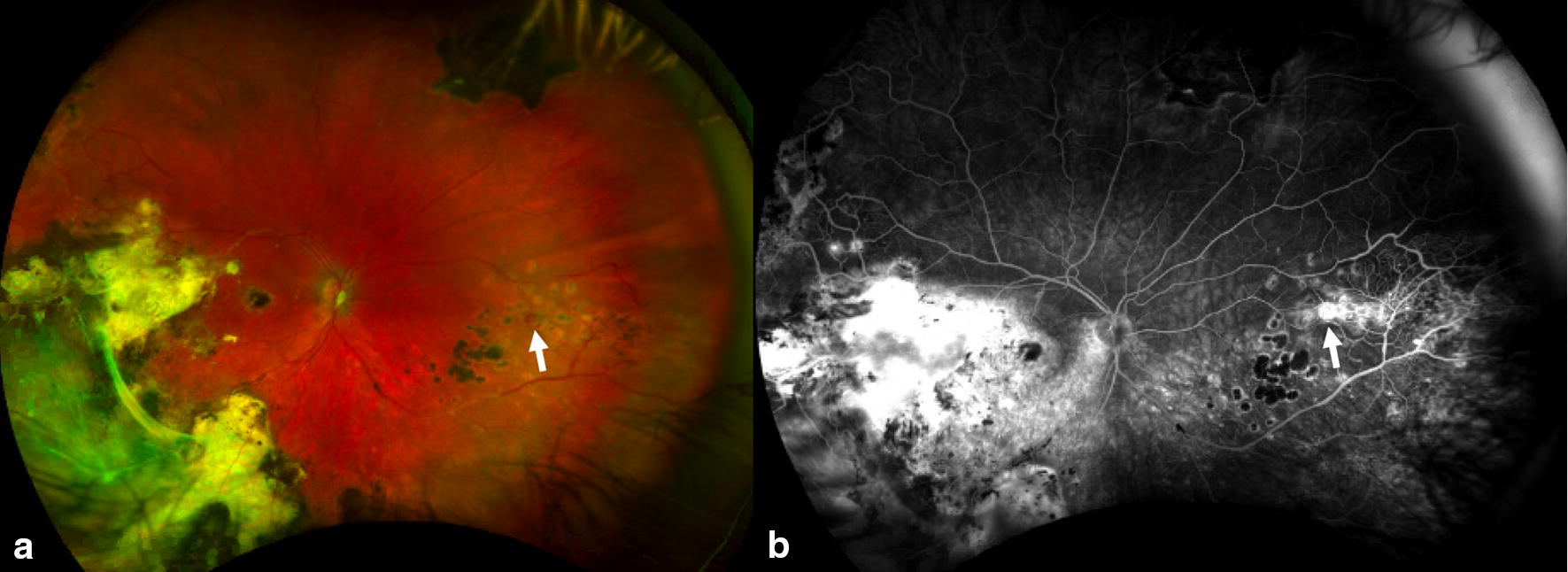
Ultra-wide field color fundus photography (**a**) and fluorescein angiography (**b**) demonstrates chorioretinal scars from previous laser treatment and an area of neovascularization nasally in patient treated for Coats disease

Three patients who received UWF-FA had been previously treated with anti-VEGF or laser photocoagulation for ROP or Coats disease. UWF-FA confirmed adequate treatment and guided the decision to observe. An example is shown in Fig. 5. Three other patients who received UWF-FA were treated with laser ablation for Coats disease based on wide field imaging findings. An example is shown in Fig. 6. One patient with newly diagnosed Coats disease required subsequent anesthesia for performance of laser photocoagulation. No patients required subsequent examination under anesthesia for diagnostic purposes.

## Discussion

This case series validates the feasibility of high quality UWF imaging in children without sedation which often can guide management. Our group previously reported 16 patients who received UWF-FA over a 5-year period [2]. In this current study, we report 14 patients who received UWF-FA over a 12-month period suggesting an increased use of the imaging modality compared to 5 years prior. The benefits of using UWF imaging have likely resulted in the increase of its use at our institution. For example, wide field fundus photography allows the physician to carefully review images with sequential comparison, and identify subtle retinal findings that may be challenging to see with examination, and monitor for progression. In addition, many conditions impacting the pediatric population such as Coats disease, ROP, FEVR have abnormalities in the peripheral retina, for which UWF imaging is particularly helpful for capturing.

We found that the number of quadrants visualized positively correlated with age, confirming that younger patients are more difficult to image. Younger patients may be less cooperative. Positioning in the chin rest and fixating on a target can be challenging. Most quadrants could be captured by obtaining serial images with positive encouragement and assistance from the parents. Several patients aged 3–4 years old were included in our study to demonstrate the feasibility of acquiring high quality images at an early age. We feel that the child friendly protocol developed in our Medical Photography department at UCLA increases the success rate of pediatric retinal imaging. Interestingly, 2 patients with nystagmus were successfully imaged in our study. Our findings corroborate those of previous case reports suggesting ultra-wide field imaging of patients with nystagmus is possible due to the rapid (0.25 s) image acquisition time [17].

We also found more quadrants of the retina were visualized during the later phases of UWF-FA.

Children often require consolation following a needle stick for the fluorescein dye resulting in an increased time to image capture and difficulty in capturing choroidal filling. In addition, the delay in positioning the patient in the chin rest results in increased artifacts in the early phases. If the early phase of angiography is of interest, intravenous access can be acquired prior to injecting fluorescein which can eliminate the transition time [2]. In addition, Ali et al. [18] described using oral fluorescein as an alternative to intravenous dye to obtain UWF-FA in children.

UWF-FAF was useful in capturing abnormal autofluorescence patterns in the macula and the periphery. Increased or attenuated autofluorescence patterns involved the periphery in 23 of 24 (96%) patients highlighting the usefulness of capturing the peripheral retina with UWF imaging. UWF-FAF has been described in case reports previously in Stargardt disease, pigmented paravenous chorioretinal atrophy, and long-chain 3-hydroxyacyl-CoA dehydrogenase retinal dystrophy [19–21]. The peripheral extent of lesions and autofluorescence patterns may be useful to diagnose heredodegenerative disorders and monitor disease activity, particularly in inflammatory conditions [22].

Lastly, in our series, the indications for obtaining imaging were quite varied as seen in Table 1 which reflects the heterogenous nature of pediatric retinal disease. In addition, in children who reported floaters or visual disturbances with a normal eye exam, UWF imaging was useful to confirm and document a normal funduscopic examination. Several studies have demonstrated that UWF is useful as a screening modality for diabetic retinopathy with a sensitivity of 94% and specificity of 100% between UWF imaging and clinical examination [23–25]. The use of UWF imaging as a screening modality can be applied to the pediatric retinal population as well.

Similarly, UWF-FA was particularly useful to confirm lack of activity or guide targeted panretinal photocoagulation in retinal vascular diseases [2, 9–11]. Moreover determining if a child who was previously treated requires additional treatment can be challenging. A negative UWF-FA, meaning one without significant ischemia or leakage, can guide the decision to observe with serial examinations and photographs and eliminate the need for an exam under anesthesia as shown in Fig. 4. On the contrary, a UWF-FA with active leakage or neovascularization as in a patient with proliferative diabetic retinopathy or Coats disease may indicate the need for additional panretinal photocoagulation (Fig. 5).

This study is limited by its retrospective nature. Another limitation of UWF-FA is that early vascular filling may not be imaged due to transition time from injection to photography. In addition, the periphery may be obscured by lash or nose artifacts. However, a strength of our study is the relatively large number of subjects included and we overcame challenges in positioning with coaching and parent involvement.

Future prospective studies can be considered to evaluate the time it takes to image subjects by age and compare UWF imaging with standard non-wide field cameras in the pediatric population. In addition, cost/benefit analysis of population screening in children using UWF technology can be considered.

## Conclusions

Our case series demonstrates that UWF imaging is a practical and useful clinical tool in the outpatient setting in the pediatric population. UWF imaging guided management and allowed for objective monitoring of disease progression and parent counseling.

## Abbreviations

EUA: examination under anesthesia
FEVR: familial exudative vitreoretinopathy
ROP: retinopathy of prematurity
UWF-FAF: ultra-wide field autofluorescence
UWF-FA: ultra-wide field fluorescein angiography
UWF: ultra-wide field
